# Supplementary material for: Rational tuning of temperature sensitivity of the TRPM8 channel
Source: EMBO Rep. 2025 Nov 14;26(24):6325–45. doi: 10.1038/s44319-025-00630-2 (PMC12715194; doi:10.1038/s44319-025-00630-2)
Supplement: Supplementary file 1 — Table EV1 [file 44319_2025_630_MOESM1_ESM.docx]

| NO. | Residue | Fold change(FC) in relative oxidation efficiency | Slope factor  Hessa et al | | Slope factor  Moon&Fleming |
| --- | --- | --- | --- | --- | --- |
| 1 | W137 | 1.25 | | 16.68 | 5.14 |
| 2 | I146 | 1.12 | | -1.14 | -5.95 |
| 3 | P159 | 0.55 | | -0.60 | -0.54 |
| 4 | W178 | 0.98 | | —— | —— |
| 5 | M189 | 0.81 | | -3.78 | -0.60 |
| 6 | Y191 | 1.05 | | 1.75 | 1.24 |
| 7 | W217 | 1.57 | | 3.24 | -1.11 |
| 8 | M242 | 0.73 | | 2.80 | -1.30 |
| 9 | N265 | 1.38 | | -3.02 | -1.22 |
| 10 | H268 | 2.21 | | -3.98 | -3.71 |
| 11 | M353 | 0.70 | | -4.67 | -3.27 |
| 12 | W380 | 1.30 | | 6.81 | 3.94 |
| 13 | K395 | 0.88 | | -2.76 | -1.36 |
| 14 | M396 | 1.21 | | 0.23 | 0.58 |
| 15 | Y410 | 0.62 | | 7.70 | 2.08 |
| 16 | W426 | 0.52 | | -8.37 | -3.96 |
| 17 | W436 | 0.62 | | 0.48 | -0.70 |
| 18 | W453 | 1.62 | | 10.46 | 4.05 |
| 19 | M462 | 0.72 | | -47.47 | -26.94 |
| 20 | Y506 | 0.72 | | 5.28 | 2.80 |
| 21 | Y516 | 0.99 | | 4.13 | 1.95 |
| 22 | W525 | 1.16 | | -0.15 | -0.55 |
| 23 | W536 | 0.76 | | 2.82 | 2.68 |
| 24 | L546 | 1.22 | | -1.72 | -1.32 |
| 25 | W567 | 1.27 | | 35.00 | 20.8 |
| 26 | W651 | 0.87 | | -28.81 | -12.02 |
| 27 | M859 | 0.78 | | 3.03 | 2.79 |
| 28 | W898 | 0.48 | | 12.56 | 12.22 |
| 29 | M911 | 1.29 | | 45.61 | 22.57 |
| 30 | F912 | 0.66 | | -28.96 | 22.21 |
| 31 | C940 | 1.27 | | —— | —— |
| 32 | M1038 | 1.42 | | 2.20 | 2.63 |
| 33 | M1059 | 1.02 | | 82.64 | 39.35 |

**Table EV1.** Fold change (FC) in relative oxidation efficiency of the residues with buried/exposed changes identified by HRF-MS. FC was calculated as the ratio of relative oxidation efficiency determined at 4℃ to the value measured at 30℃. The slope factor was calculated by fitting SCH and ΔH values to a linear function for each site with buried/exposed changes. The hydrophobicity scale of SCH was determined by Hessa et al and Moon et al, respectively.
