# Supplementary material for: Rational tuning of temperature sensitivity of the TRPM8 channel
Source: EMBO Rep. 2025 Nov 14;26(24):6325–45. doi: 10.1038/s44319-025-00630-2 (PMC12715194; doi:10.1038/s44319-025-00630-2)
Supplement: Supplementary file 2 — Table EV2 [file 44319_2025_630_MOESM2_ESM.docx]

| NO. | Residue | I*_cold_* / I*_menthol_*  (+ 80 mV) | cold activation thresholds  (+ 80 mV) | cold activation thresholds  (- 80 mV) |
| --- | --- | --- | --- | --- |
| 1 | WT | 0.81 ± 0.02 | 25.88 ± 0.45°C | 22.12 ± 0.73°C |
| 2 | W137H | 0.13 ± 0.01 | 27.55 ± 0.40°C | Too small to quantify |
| 3 | W453H | 0.28 ± 0.02 | 24.60 ± 0.27°C | Too small to quantify |
| 4 | W462I | 0.21 ± 0.01 | 28.45 ± 1.42°C | 26.61 ± 0.36°C |
| 5 | W651L | 0.40 ± 0.02 | 22.47 ± 1.65°C | 21.18 ± 1.54°C |

**Table EV2.** Electrophysiological characterization of WT and mutants that alter temperature sensitivity. Summary of cold/menthol current ratios at +80 mV and thermal activation thresholds measured at ±80 mV holding potentials for mutations at residues exhibiting high thermosensitivity (mean ± s.e.m.).
