# Supplementary material for: Rational tuning of temperature sensitivity of the TRPM8 channel
Source: EMBO Rep. 2025 Nov 14;26(24):6325–45. doi: 10.1038/s44319-025-00630-2 (PMC12715194; doi:10.1038/s44319-025-00630-2)
Supplement: Supplementary file 3 — Table EV3 [file 44319_2025_630_MOESM3_ESM.docx]

**Table EV3.** Changes in energy measured from MD simulation of TRPM8. Changes were calculated as energy values at 4℃ minus those at 30℃ (mean ± s.e.m.).

| Interacting components | Electrostatic energe changes (kcal/mol) | Electrostatic energe changes scaled by dielectric coefficient of 80 (kcal/mol) | vdw energy changes (kcal/mol) |
| --- | --- | --- | --- |
| Protein-water interactions in: |  |  |  |
| MHR1-3 | -2432.9 ± 58.7 | -30.4 ± 0.7 | -146.8 ± 4.3 |
| MHR4 | -1725.2 ± 25.6 | -21.6 ± 0.3 | -125.8 ± 2.3 |
| The rest | -2625.0 ± 30.3 | -32.8 ± 0.4 | -215.1 ± 3.7 |
| Whole TRPM8 protein | -6783.1 ± 63.3 | -84.8 ± 0.8 | -487.7 ± 4.8 |
|  |  |  |  |
| Protein-lipid interactions | 451.6 ±10.7 | 5.6 ± 0.1 | 7.55 ± 3.1 |
| Intra-protein interactions | 2233.4 ± 24.3 | 27.9 ± 0.3 | -211.6 ± 4.6 |
