# Supplementary material for: Rational tuning of temperature sensitivity of the TRPM8 channel
Source: EMBO Rep. 2025 Nov 14;26(24):6325–45. doi: 10.1038/s44319-025-00630-2 (PMC12715194; doi:10.1038/s44319-025-00630-2)
Supplement: Supplementary file 4 — Table EV4 [file 44319_2025_630_MOESM4_ESM.docx]

**Table EV4.** Changes in water counts measured from MD simulation of TRPM8. Changes were calculated as water counts at 4℃ minus those at 30℃. Water molecules within 4.5 Å of the sidechain in a residue were counted (mean ± s.e.m.). The values highlighted in blue in the table represent the number of water molecules surrounding the cold-sensitive amino acids shown in Fig. 2G.

| **NO.** | **Residue** | **Change in water counts**  **(4°C-30°C)** |
| --- | --- | --- |
| 1 | W137 | 8.26 ± 0.24 |
| 2 | I146 | 1.66 ± 0.25 |
| 3 | P159 | 0.05 ± 0.19 |
| 4 | W178 | 4.97 ± 0.15 |
| 5 | M189 | 0.96 ± 0.20 |
| 6 | Y191 | 6.04 ± 0.24 |
| 7 | W217 | 0.56 ± 0.12 |
| 8 | M242 | 3.41 ± 0.12 |
| 9 | N265 | 2.57 ± 0.19 |
| 10 | H268 | -1.74 ± 0.15 |
| 11 | M353 | 0.00 ± 0.28 |
| 12 | W380 | -0.17 ± 0.18 |
| 13 | K395 | -1.76 ± 0.25 |
| 14 | M396 | 0.15 ± 0.11 |
| 15 | Y410 | 5.00 ± 0.17 |
| 16 | W426 | 7.99 ± 0.20 |
| 17 | W436 | 2.18 ± 0.14 |
| 18 | W453 | 6.50 ± 0.17 |
| 19 | M462 | 0.40 ± 0.15 |
| 20 | Y506 | 13.18 ± 0.24 |
| 21 | Y516 | -1.93 ± 0.23 |
| 22 | W525 | -4.56 ± 0.08 |
| 23 | W536 | 9.74 ± 0.19 |
| 24 | L546 | -0.44 ± 0.20 |
| 25 | W567 | 5.90 ± 0.01 |
| 26 | W651 | 1.67 ± 0.19 |
| 27 | M859 | -1.42 ± 0.14 |
| 28 | W898 | -0.68 ± 0.14 |
| 29 | M911 | -0.09 ± 0.05 |
| 30 | F912 | 0.90 ± 0.15 |
| 31 | C940 | 10.16 ± 0.26 |
| 32 | M1038 | 4.97 ± 0.28 |
| 33 | M1059 | 0.93 ± 0.12 |
