# Supplementary material for: Rational tuning of temperature sensitivity of the TRPM8 channel
Source: EMBO Rep. 2025 Nov 14;26(24):6325–45. doi: 10.1038/s44319-025-00630-2 (PMC12715194; doi:10.1038/s44319-025-00630-2)
Supplement: Supplementary file 5 — Table EV5 [file 44319_2025_630_MOESM5_ESM.docx]

**Table EV5.** Cold and menthol activation of TRPM8 mutants at residue W137. Temperature was decreased to below 10℃ to activated TRPM8 mutants. A saturating concentration of 2 mM menthol was used in ligand activation (mean ± SEM). The values highlighted in blue in the table represent the amino acids used for calculating the enthalpy change (ΔH) of cold activation.

| NO. | Mutant | Cold Activation | Menthol Activation |
| --- | --- | --- | --- |
| 1 | W137R | NO | NO |
| 2 | W137H | YES | YES |
| 3 | W137K | NO | NO |
| 4 | W137D | NO | NO |
| 5 | W137E | YES | YES |
| 6 | W137S | NO | NO |
| 7 | W137T | NO | NO |
| 8 | W137N | Too small to quantify | YES |
| 9 | W137Q | Too small to quantify | YES |
| 10 | W137C | NO | NO |
| 11 | W137G | NO | NO |
| 12 | W137P | NO | NO |
| 13 | W137A | NO | NO |
| 14 | W137V | NO | NO |
| 15 | W137I | NO | NO |
| 16 | W137L | YES | YES |
| 17 | W137M | YES | YES |
| 18 | W137F | YES | YES |
| 19 | W137Y | YES | YES |
