# Supplementary material for: Rational tuning of temperature sensitivity of the TRPM8 channel
Source: EMBO Rep. 2025 Nov 14;26(24):6325–45. doi: 10.1038/s44319-025-00630-2 (PMC12715194; doi:10.1038/s44319-025-00630-2)
Supplement: Supplementary file 16 — Expanded View Figures [file 44319_2025_630_MOESM16_ESM.pdf]

Expanded View Figures

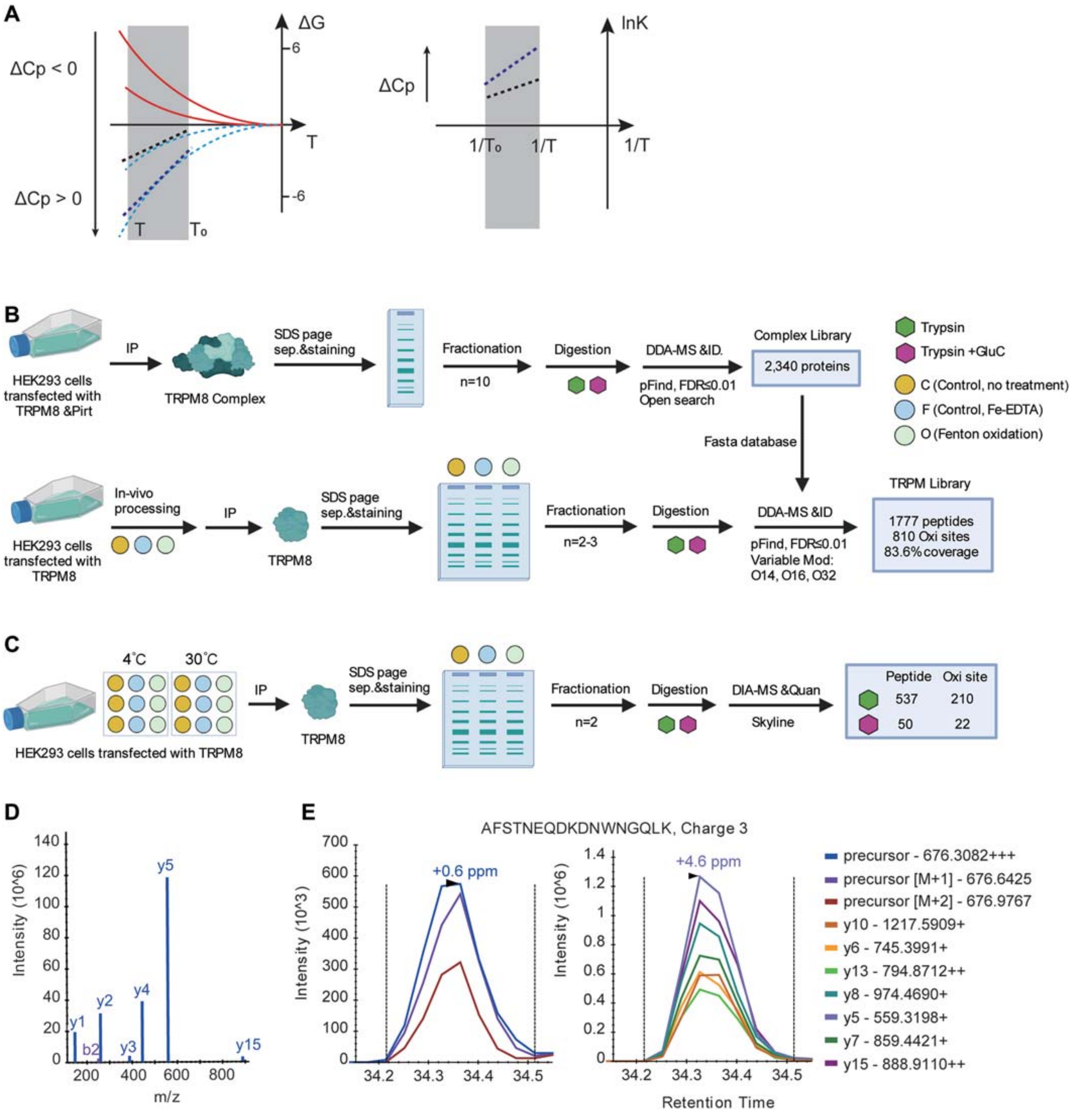

Figure EV1. Thermodynamic analysis and spectral library generation for TRPM8 oxidation (Related to Fig. 1).

(**A**) Residues contributing to ΔCp were mutated to be either polar or hydrophobic. The magnitude of ΔCp influences the degree of curvature, with larger absolute values producing greater curvature. The van't Hoff equation was used to determine ΔH and ΔS from a linear fit of lnK versus 1/T. As the absolute value of ΔCp increases, the slope of the linear fit becomes steeper, resulting in higher absolute values of ΔH and ΔS (gray region). (**B**, **C**) The workflow for TRPM8 spectral library generation and oxidation site determination and quantification. DIA data-independent acquisition. (**D**) Peptide fragmentation spectra from the spectral library. (**E**) Extracted ion chromatogram (XIC) groups (left: MS1, right: MS2) from the quantification results of an example peptide AFSTNEQDKDNWNGQLK.

 © The Author(s)

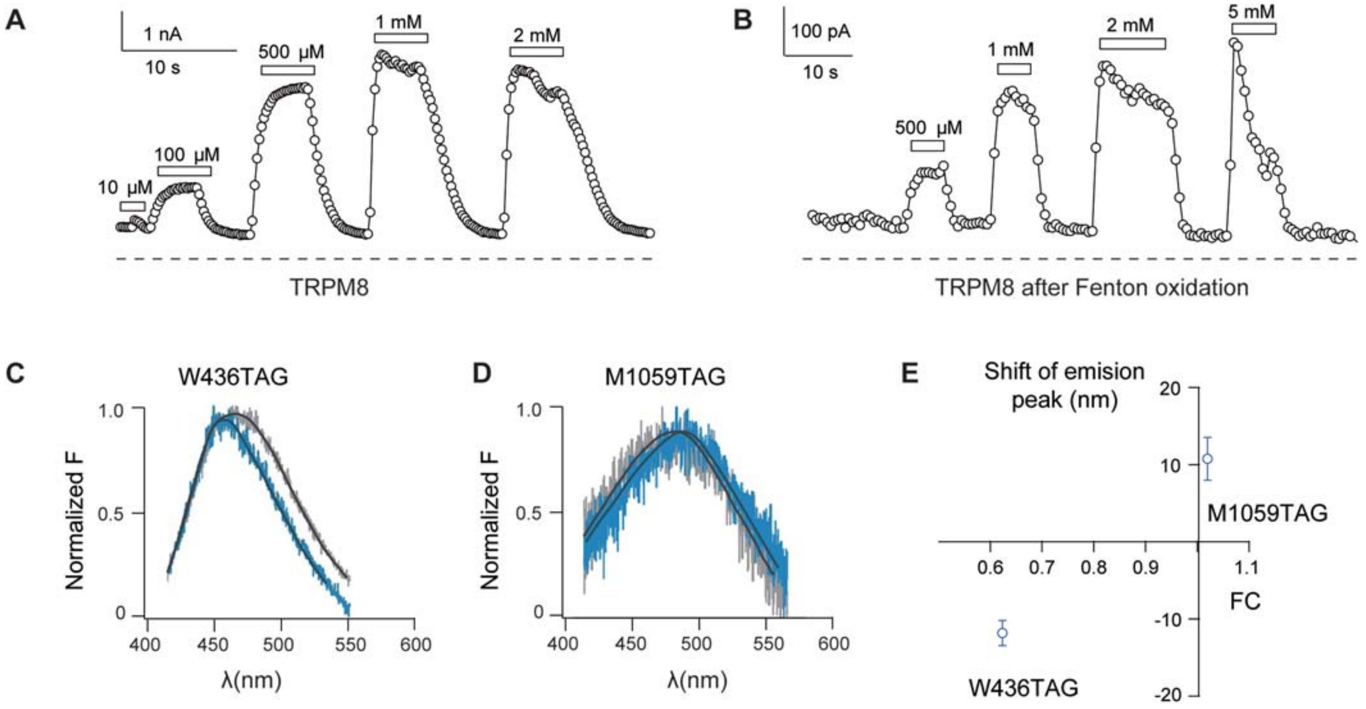

Figure EV2. Effects of oxidation on TRPM8 channel activity and the properties of ANAP-incorporated mutants.

(Related to Fig. 1). (**A**, **B**) Representative whole-cell current recordings of the TRPM8 channel activated by menthol before and after Fenton oxidation. (**C**, **D**) Representative emission spectra of ANAP incorporated at residue W436 and M1059, respectively. Emission spectra in gray and blue were measured at either 30 or 4 °C, respectively. (**E**) Comparison of shifts in emission spectra peak of ANAP incorporated at residue W436 and M1059 with their FC values measured from HRF-MS. (The y-axis represents the emission spectra peak of ANAP; $n = 3$ biological replicates; data were presented as mean ± s.e.m.).

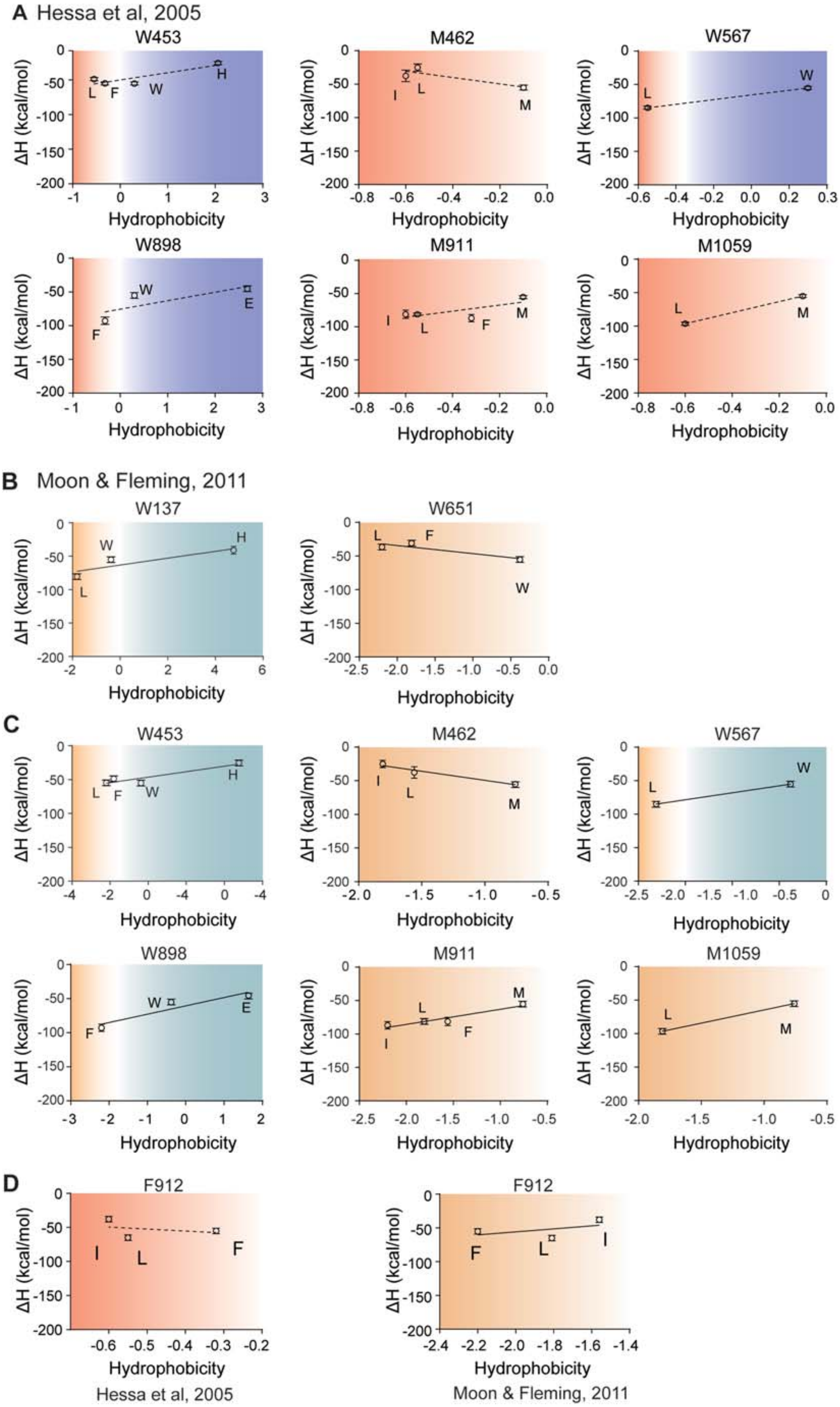
A Hessa et al, 2005
W453
M462
W567
W898
M911
M1059
B Moon & Fleming, 2011
W137
W651
C
W453
M462
W567
W898
M911
M1059
D
F912
F912
ΔH (kcal/mol)
Hydrophobicity
Hessa et al, 2005
Moon & Fleming, 2011

 © The Author(s)

Figure EV3. Correlation of residue SCH and ΔH in TRPM8 under various SCH hydrophobicity scales during cold activation.

(Related to Fig. 2). (**A–C**) Correlation between SCH and ΔH values for TRPM8 residues with changes in FC values during cold activation. The hydrophobicity values shown on the x-axis in (**A**) were based on the SCH hydrophobicity scale reported by Hessa et al, whereas those in Panels (**B**) and (**C**) were based on the scale reported by Moon et al. (**D**) The F912 mutation was excluded due to substantial differences in the hydrophobicity scales reported by Hessa et al and Moon et al.

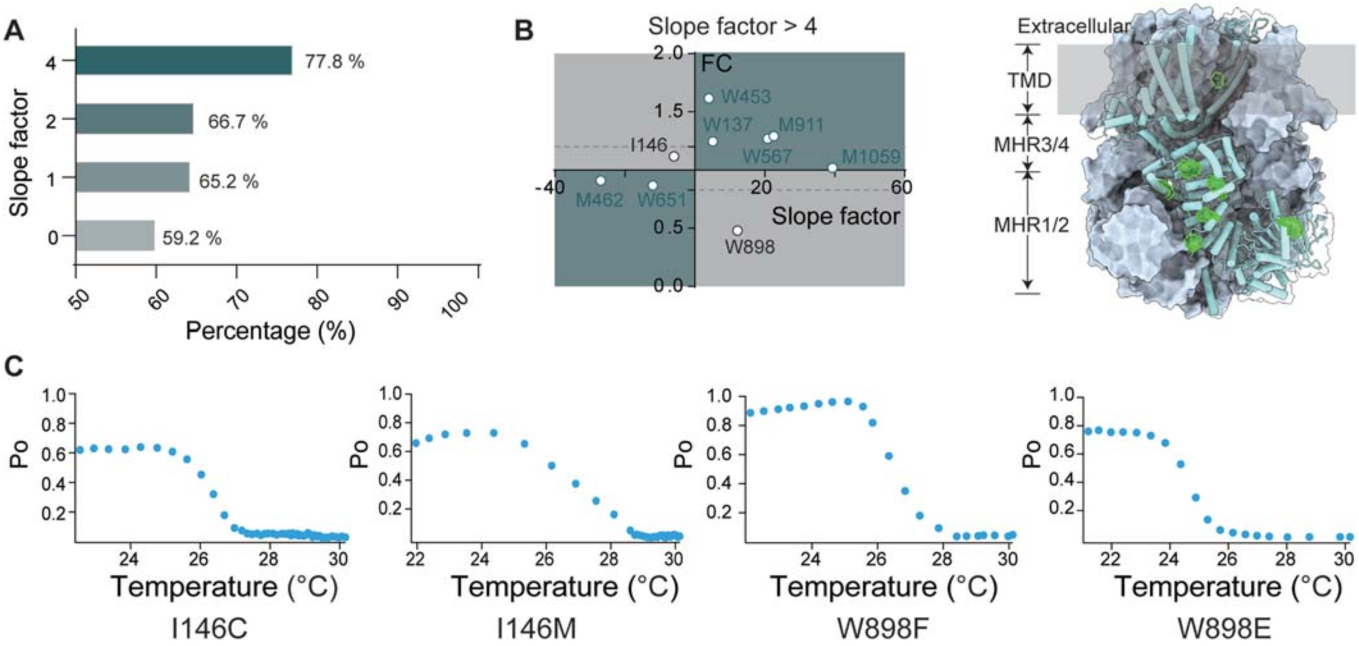

Figure EV4. Relationship between residue SCH and ΔH in TRPM8 cold sensitivity.

(Related to Fig. 2). (**A**) The proportion that aligns with the temperature sensitivity hypothesis increased as the slope factor increased. The slope factor was calculated by fitting SCH and ΔH values to a linear function for each site with buried/exposed changes. The hydrophobicity scale of SCH was determined by Moon et al. The data were then grouped into five classes, with absolute slope factor values greater than 0, 1, 2, and 4, respectively. The x-axis represents the proportion that aligns with the temperature sensitivity hypothesis. (**B**) Correlation between SCH and ΔH values for TRPM8 residues with changes in FC values during cold activation. The hydrophobicity scale of SCH was determined by Moon et al. The slope factor was plotted against the FC value of the corresponding site. The first and third quadrant, where the sites followed the predictions from the water–protein interaction hypothesis of cold sensing, were shaded in green. The second and fourth quadrant, where the sites did not follow the predictions from the water–protein interaction hypothesis of cold sensing, were shaded in gray. The sites located in the first and third quadrant were mapped onto the cryo-EM structure of TRPM8 with their sidechains shown in green. (**C**) Representative whole-cell current recordings of TRPM8 mutants that deviated from the hypothesis exhibited cold-activated properties.

 © The Author(s)

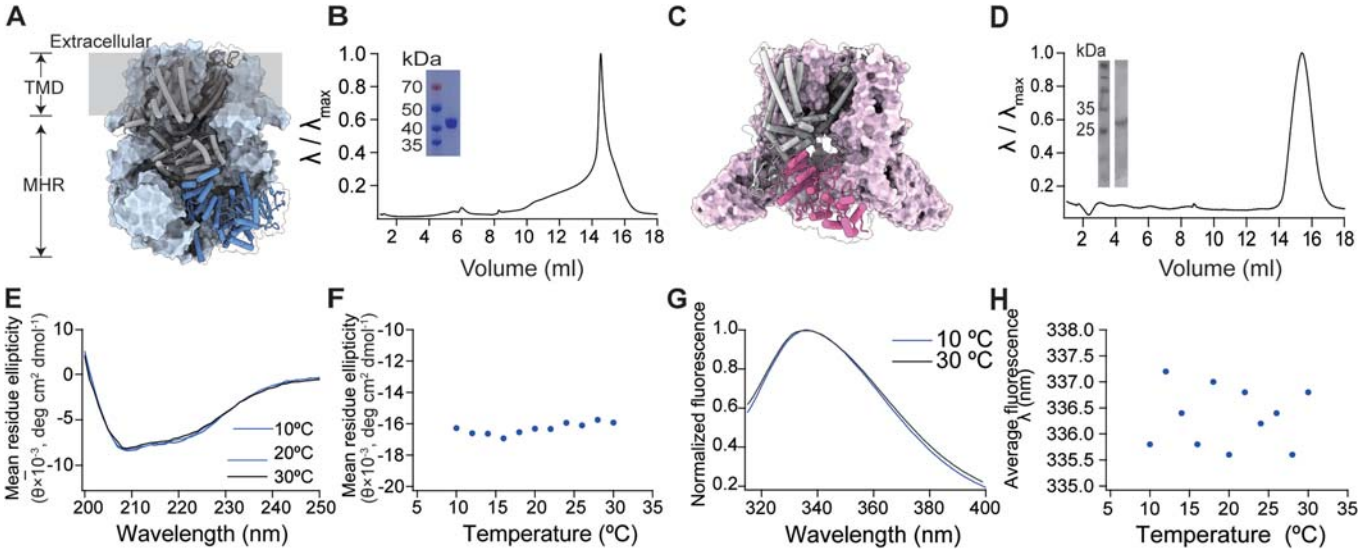

Figure EV5. Structural and biophysical characterization of TRPM8 MHR1-3 and TRPV2 ARD domains.

(Related to Fig. 3). (**A**) The location of MHR1-3 domains (colored in blue) in TRPM8. (**B**) Size-exclusion chromatography of the protein of MHR1-3 domains on Superose 6 (GE Healthcare) and SDS-PAGE. (**C**) The location of ARD domains (colored in pink) in TRPV2. (**D**) Size-exclusion chromatography of the protein of ARD on Superose 6 (GE Healthcare) and SDS-PAGE. (**E**) Representative CD spectra of ARD in TRPV2. (**F**) The temperature dependence of the CD spectra. (**G**, **H**) Representative intrinsic tryptophan emission spectra of ARD in TRPV2 and temperature dependence of intrinsic tryptophan emission peaks, respectively.
